# Carbohydrates great and small, from dietary fiber to sialic acids: How glycans influence the gut microbiome and affect human health

**DOI:** 10.1080/19490976.2020.1869502

**Published:** 2021-02-21

**Authors:** Joanna K Coker, Oriane Moyne, Dmitry A. Rodionov, Karsten Zengler

**Affiliations:** a Department of Pediatrics, University of California, San Diego, La Jolla, USA; b Sanford Burnham Prebys Medical Discovery Institute, La Jolla, USA; c A.A. Kharkevich Institute for Information Transmission Problems, Russian Academy of Sciences, Moscow, Russia; d Center for Microbiome Innovation, University of California, San Diego, La Jolla, USA; e Department of Bioengineering, University of California, San Diego, La Jolla, USA

**Keywords:** Carbohydrates, glycans, gut microbiome, diet, fiber, sialic acids, neu5gc, mucin-linked *O*-glycans, human milk oligosaccharides

## Abstract

Gut microbiome composition depends heavily upon diet and has strong ties to human health. Dietary carbohydrates shape the gut microbiome by providing a potent nutrient source for particular microbes. This review explores how dietary carbohydrates in general, including individual monosaccharides and complex polysaccharides, influence the gut microbiome with subsequent effects on host health and disease. In particular, the effects of sialic acids, a prominent and influential class of monosaccharides, are discussed. Complex plant carbohydrates, such as dietary fiber, generally promote microbial production of compounds beneficial to the host while preventing degradation of host carbohydrates from colonic mucus. In contrast, simple and easily digestible sugars such as glucose are often associated with adverse effects on health and the microbiome. The monosaccharide class of sialic acids exerts a powerful but nuanced effect on gut microbiota. Sialic acid consumption (in monosaccharide form, or as part of human milk oligosaccharides or certain animal-based foods) drives the growth of organisms with sialic acid metabolism capabilities. Minor chemical modifications of Neu5Ac, the most common form of sialic acid, can alter these effects. All aspects of carbohydrate composition are therefore relevant to consider when designing dietary therapeutic strategies to alter the gut microbiome.

## Introduction

The human gut microbiome is defined as the sum of genomic DNA of all microbes inhabiting this environment. With up to a hundred times more bacterial genes than human genes in the human body, including an especially high number of microbes in the gut, the microbial communities in our body are crucial to human life and play a key role in human development and homeostasis.^1^ As in every natural ecosystem, bacteria in the human gut influence the surrounding environment of their host. The human gut microbiota is involved in many essential host functions, such as the processing of nutrients to bioactive molecules like neurotransmitters, vitamins, and fatty acids and protection from pathogens.^2^ One of the most well-known examples of this is the breakdown of non-digestible carbohydrates found in plants. As humans do not have the metabolic capability to degrade these complex glycans in the gastrointestinal tract, they reach the colon to be fermented by gut bacteria and lead to the production of short-chain fatty acids (SCFAs), which participate in the acidification of the digestive tract.^3^ Through these and other similar processes, the human gut microbiota has a major impact on the host’s physiology in health and disease.

In addition to a greater number of genes and metabolic capabilities than the human genome, the composition of the gut microbiome is also highly malleable.^1^ Diet surpasses the role of host genetics in shaping the gut microbiome through modification of the nutritional environment of the bacteria populating the gut.^4–8^ Given the influence of the gut microbiome in human health, the ability to alter this microbiome through dietary changes indicates that promoting a healthy microbiome has great potential to improve human well-being and disease prevention and control. Glycans (i.e. carbohydrates) are of major importance in determining the gut microbiome composition.^9^ Glycans come in many forms, from long polysaccharide chains that humans are unable to digest (e.g. cellulose, pectins, resistant starch), to oligosaccharide chains attached to proteins and lipids, to individual mono – and disaccharides, such as glucose, lactose, or sialic acids.^9^ In this review, we detail how dietary glycans can shape the structure and function of the human gut microbiota and the impact this has on human diseases. We start with an overview of the broad impacts of carbohydrates on gut microbiota composition and metabolic activity. We then focus on the role of sialic acids, a specific monosaccharide class, in shaping the gut microbiome. Sialic acids are a prominent component of the mammalian glycosylation system, and their interactions with the human immune system make their impact on the gut microbiome of particular interest. This review of sialic acids, the gut microbiome, and impacts on health will summarize recent research and suggest directions for future studies.

## Broad impact of carbohydrates on gut microbiome structure and function

Human studies have repeatedly demonstrated that dietary changes modify the relative abundance of major gut bacterial groups in a rapid and reversible manner.^10,11^ For example, low-carbohydrate, weight-loss, and animal-based diets reduce the proportion of the butyrate-producing phyla *Firmicutes* and *Actinobacteria*,^11–13^ while high animal product consumption increases the proportion of *Bacteroidetes* and specific *Proteobacteria* like *Bilophila wadsworthia* in the human gut.^11^ Lifestyle urbanization and Westernization are key factors influencing dietary behavior, with subsequent impacts on the gut microbiome and potential harmful effects on human health.^14^ A rural diet, typically rich in host-indigestible carbohydrates like fiber, is associated with a higher abundance of *Prevotella* and *Xylanibacter spp*., while an urbanized diet, which generally contains more saturated fat and protein from animals, is associated with an increase of *Bacteroides spp*. and a decrease of overall microbiome gene diversity.^15–17^ Interestingly, those *Bacteroides*-dominated, less diverse gut communities are associated with a higher incidence of obesity and metabolic syndrome.^18^ The loss of diversity and shift from a *Prevotella* – to *Bacteroides*-dominated microbiome has been observed in non-Western immigrants as early as 9 months after moving to the USA.^19^ These data demonstrate the plasticity of the human gut microbiota in response to dietary carbohydrate changes and the potential impact of these changes on human health.

Many studies examine the impact of plant carbohydrates in particular on the gut microbiome. Diets with high resistant starch intake have been associated with increased relative abundance of *Firmicutes* and particularly *Ruminococcaceae* family members, while resistant potato starch specifically has been associated with increased *Bifidobacterium* genera and wheat bran has been associated with increased *Lachnospiraceae* family members.^10,12,20^ A recent study also demonstrated rapid modifications of the gut microbiota in mice fed raw versus cooked plant products, due to the improvement of starch digestibility and degradation of plant-derived compounds during the cooking process. Similar observations have been made in the human population, showing that everyday nutritional habits can influence the gut microbiota.^8^

**Figure 1.**
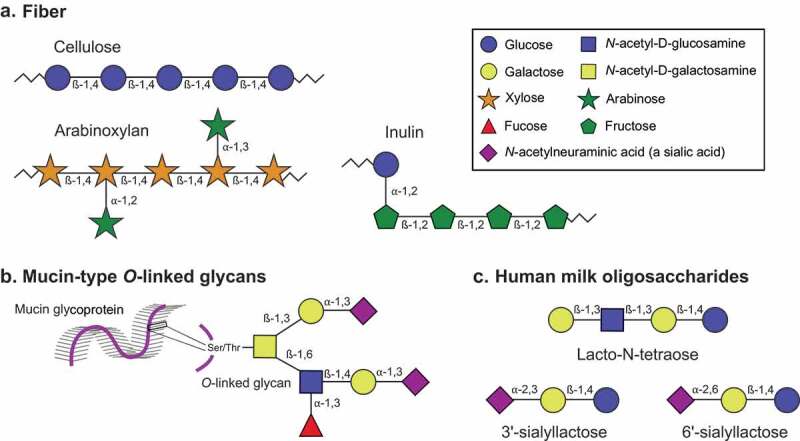
Structural composition of the main poly – and oligosaccharides discussed in this review. Monosaccharide symbols are represented as in the Symbol Nomenclature for Glycans (assignments for this figure provided in the box).^22,23^ The numbers between monosaccharides represent the glycosidic linkage. A) Fiber is a general classification encompassing many types of dietary polysaccharides. Examples of the polysaccharides cellulose, arabinoxylan, and inulin are provided here. B) Mucin-type *O*-linked glycans are host glycans linked to serine or threonine (Ser/Thr) residues on mucin proteins. Like most mammal-derived glycans, they are often tipped with sialic acids such as *N*-acetylneuraminic acid. An example structure is shown; many other monosaccharide and linkage compositions are possible. C) Human milk oligosaccharides (HMOs) are short oligosaccharides found in human breast milk. HMOs are composed of a lactose base (a disaccharide of glucose and galactose) with additional monosaccharides such as *N*-acetyl-D-glucosamine, the sialic acid *N*-acetylneuraminic acid, or fucose attached. Three example structures are shown

Within plant carbohydrates, dietary fiber is one of the most heavily studied groups. Dietary fiber is generally defined as edible carbohydrate polymers, mostly from plants and edible fungi, that are not digestible by human enzymes.^21^ Examples include inulin, dextrin, pectin, cellulose, resistant starch, arabinoxylans, and chitin (**Figure 1A**).^24^ Dietary fiber exists in soluble and insoluble forms, although some polymers can be soluble or insoluble depending on conditions like cooking or food processing.^21^ Although human metabolism cannot digest fiber, the gut microbiome often contains many enzymes capable of degrading these polymers and utilizing the sugars released for nutrition or other metabolic processes.

**Figure 2.**
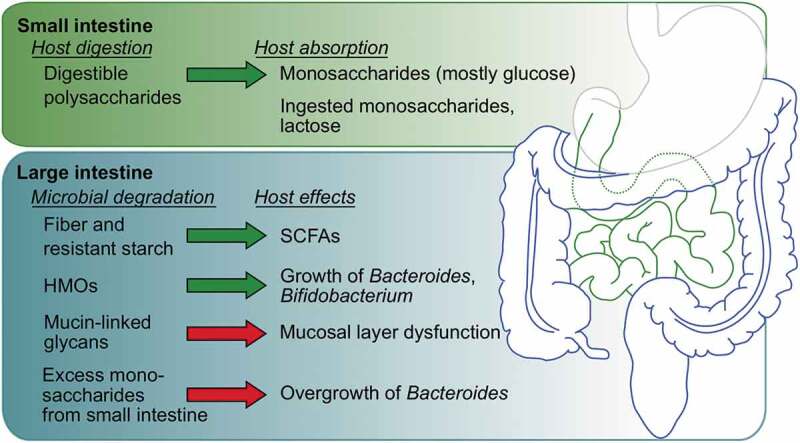
Broad overview of carbohydrate digestion and host effects discussed in this review; other effects are also possible for each glycan. Generally beneficial and detrimental effects are represented by green and red arrows, respectively. Most host digestion and nutrient absorption occurs in the small intestine. Microbes conduct most of the nutrient degradation that occurs in the large intestine, with varying effects on host health and function

Fiber passes mostly undigested through the small intestine and is fermented in the colon by gut bacteria, leading to the production of SCFAs (**Figure 2**).^25^ The SCFAs acetate, propionate, and butyrate are the main metabolites produced during microbial fiber fermentation, and they have multiple beneficial effects on the host.^14^ Once produced in the colon, SCFAs are rapidly absorbed by host epithelial cells, where the great majority are directly used as an energy source. SCFAs that are not metabolized by the gut epithelium (estimated as <10% of total SCFAs produced)^26,27^ are then transported through the portal circulation to the liver, where they can be incorporated by hepatocytes and used as energy substrates or for the synthesis of glucose, cholesterol, and fatty acids.^28^

A small fraction of the initial SCFAs will reach the main blood circulation and have systemic effects, particularly on the immune system.^28^ Notably, SCFAs downregulate the production of pro-inflammatory cytokines by colonic macrophages^29^ and promote the differentiation of naive CD4 + T cells into immunosuppressive regulatory T cells (Treg),^30,31^ by binding to G-protein coupled receptors^32^ or by inhibiting histone deacetylases.^29,33,34^ SCFAs derived by the gut microbiota from dietary fiber thus participate in the homeostasis of the immune response, with a demonstrated protective effect against inflammatory diseases, such as multiple sclerosis (MS),^33^ inflammatory bowel disease (IBD),^31^ and allergic asthma,^35^ as well as other pathologies, such as infection^36,37^ and carcinogenesis.^16^ Colorectal cancer (CRC) is also known to be linked with a gut microbiota dysbiosis characterized by decreased microbial diversity^38^ and an under-representation of SCFA-producing bacteria.^39^ A high-fiber dietary intake is associated with a lower risk of CRC,^40^ while patients with CRC precursor lesions tend to have lower fiber dietary intake than controls.^41^

However, not all studies have shown universal benefits from fiber intake. In a recent study, Singh et al. supplemented the diet of toll-like receptor 5 (TLR5)-deficient mice with fermentable fibers for 6 months, with the goal of demonstrating the beneficial effect of such a diet on metabolic syndrome. While the authors observed some of the expected effects (reduction of adiposity, amelioration of glycemic control), they also observed that purified fiber supplementation induced icteric hepatocellular carcinoma in 40% of the TLR5-deficient mice.^42^ These studies indicate that dietary supplementation with such purified compounds may have a negative effect on some individuals, and that large-scale enrichment of processed food with purified prebiotic fiber should be taken with great caution.^43^ For a more detailed discussion of the gut microbiota and specific health effects of dietary fiber, we refer the reader to ref. 42.^44^

Mono – and disaccharide dietary sugars can affect gut microbiome composition, with potential effects on human health. Fructose and glucose have been demonstrated to specifically inhibit gut colonization by *Bacteroides thetaiotaomicron*, a mammal gut symbiont associated with lean and healthy individuals, by silencing the Roc (regulator of colonization) protein, which promotes competitive colonization in gnotobiotic mice.^45^ High fructose intake has also been associated with development of nonalcoholic fatty liver disease (NAFLD) in humans^46^ and mouse models.^47,48^ The gut microbiome in general plays a causal role in NAFLD development in mouse models,^49^ and several studies have established correlations between NAFLD and altered abundance of taxa, such as *Bifidobacterium*,^50^ *Lactobacillus*,^50,51^ *Bacteroides*, and *Ruminococcus*.^52^ Supplementation of *Lactobacillus rhamnosus* in the gut microbiome of mice fed a high-fructose diet to induce NAFLD resulted in decreased liver inflammation and NAFLD disease development.^51^ This finding highlights the potential regulatory effects of dietary sugars in the small intestine on gut colonization by beneficial microbes. Later in this review, we will discuss in detail recent research on the effects of sialic acids, a biologically important class of monosaccharides, on the gut microbiome and host health.

## Interplay of dietary fiber and host mucins in the gut microbiome

Although dietary glycans make up the majority of nutrients the gut microbiota consumes, restriction of carbohydrates like fiber from the diet can push microbes to consume glycans produced by the host instead.^53^ The colon contains a mucus gel layer composed of two parts: a loose luminal outer layer and a dense mucosal inner layer.^54^ The mucosal layer is composed mainly of host mucin proteins with regions of extensive *O*-glycosylation (forming up to 80% of the total mucin mass) (**Figure 1B**).^55^ Although microbes do not penetrate the dense inner layer in healthy subjects,^37,56^ microbial degradation of the outer layer is thought to be a normal part of mucin turnover and regeneration.^57^ For a review of how gut microbiota interact with and degrade the colonic mucosal layer, we direct the reader to ref. 56.^58^ Here we focus on how diet can alter the careful balance between gut microbiota and the host mucosal layer.

The section above discussed how the presence of complex polysaccharides, such as fiber, in the diet strongly affects the gut microbiome composition. Many studies have shown that fiber ingestion increases abundance of colonic bacteria capable of fermenting fiber to SCFAs,^7,10,13,20^ with increased diversity of plant carbohydrates believed to support greater community diversity.^59^ Conversely, several studies have shown that a lack of dietary fiber can push bacterial metabolism away from fiber degradation to mucin degradation. Some organisms (e.g. *Bacteroides thetaiotaomicron*) degrade both fiber and mucins and shift their metabolism to mucin degradation when dietary complex polysaccharides are scarce.^53,60^ Other organisms (e.g. *Akkermansia muciniphila*) are able to degrade mucins but not fiber and experience expansion of their populations upon complex polysaccharide scarcity.^37,61,62^

Excessive mucin degradation is associated with increased intestinal inflammation^63,64^ and increased penetration of bacteria into the dense mucosal mucus layer.^65^ In gnotobiotic mice mono-colonized with *B. thetaiotaomicron*, a diet lacking complex polysaccharides (including fiber) resulted in a thinner colonic mucus layer, an increased proximity of colonic microbes to the gut epithelium, and increased expression of the inflammatory marker REG3β.^60^ Similarly, it has been shown that dietary fiber deprivation increased the abundance of mucus-degrading bacteria like *A. muciniphila* and *Bacteroides caccae* in mice, subsequently leading to an alteration of the intestinal barrier and higher susceptibility to mucosal pathogens.^37,60^ Demonstrating the specific and essential role of the gut microbiome in mucus changes, antibiotic-treated mice fed a low-fiber Western diet but transplanted weekly with gut microbiota from mice fed a high-fiber chow diet had significantly lower mucus penetrability and higher mucus growth than mice transplanted with gut microbiota from Western diet-fed mice.^66^ These studies indicate a lack of dietary fiber leads to changes in the gut microbiome that promote dysfunction and increased microbial penetrability of the inner colonic mucus layer.

On the other hand, a recent study suggests potentially beneficial roles of microbial mucus metabolism in ulcerative colitis (UC). Certain organisms are capable of producing the SCFA *n*-butyrate from mucin degradation,^67^ and *n*-butyrate as well as mixed SCFAs have been shown to reduce colon inflammation in UC.^68,69^ Yamada et al.^67^ found decreased mucinase activity and decreased levels of *n*-butyrate in the stool of UC patients, but a significantly higher *O*-glycan-to-mucin protein ratio. Hypothesizing a deficiency in mucin *O*-glycan utilization by gut microbiota, the authors assessed the impact of feeding mice a mucin-enriched diet. After 3 weeks, they observed an increased α-diversity; increased relative abundance of *Akkermansia, Allobaculum*, and *Bacteroidales S24-7*; increased cecal SCFAs; and increased colonic Treg and IgA^+^ plasma cells.^67^ In the setting of UC, mucin degradation may therefore be an important physiologic process to promote.

## Impact of the monosaccharide sialic acid on gut microbiome structure and function

**Figure 3.**
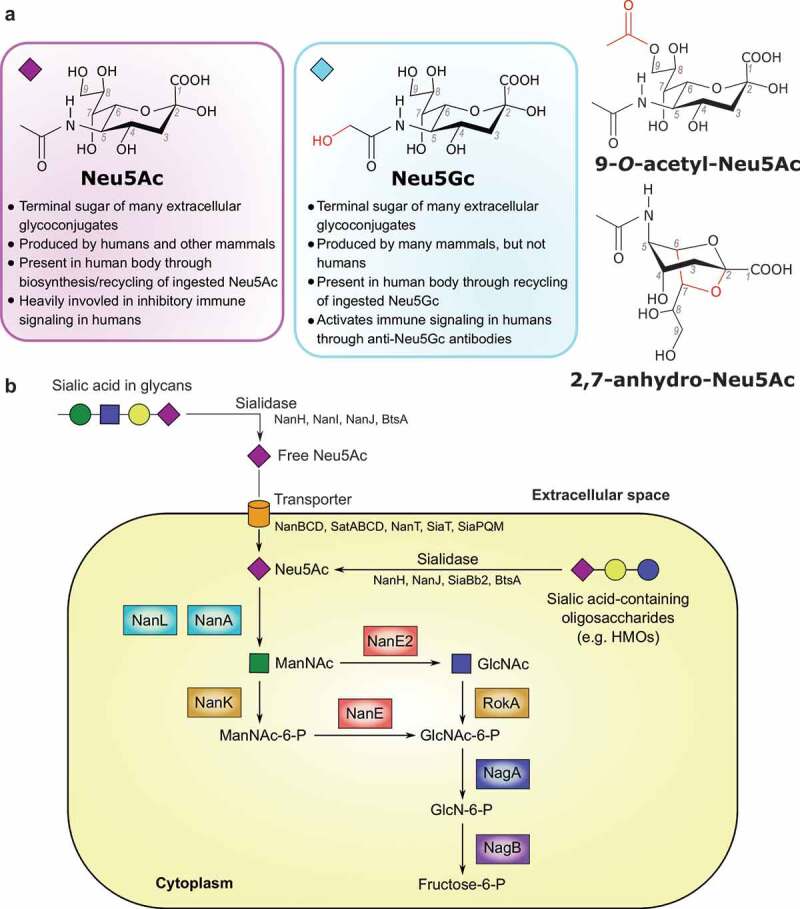
A) Structures of the sialic acids discussed in this review. Changes from the Neu5Ac structure are shown in red. Differences between Neu5Ac and Neu5Gc with regards to human physiology are listed below those structures. Diamonds by Neu5Ac and Neu5Gc structures depict the Symbol Nomenclature for Glycans symbol for each. B) Schematic of general Neu5Ac metabolism in bacterial cells. Steps with multiple characterized enzymes (sialidase and transporter) have protein names listed below the general enzyme name. Steps with one well-characterized enzyme have the enzyme name next to the arrow. The exception is the conversion of Neu5Ac to ManNAc, which has 2 well-characterized enzymes. Enzymes are color-coded based on function. References for enzyme functions: NanAEE2KL, RokA, NagAB;.^74^ NanHIJ;^75^ NanBCD;^76^ NanT;^77^ SiaBb2;^78^ BtsA;^79^ SatABCD;^80^ SiaT;^81^ SiaPQM.^82^

Thus far, we have discussed the impact of broad dietary glycan classes on the gut microbiome and host health, including how lack of fiber promotes microbial degradation of host mucus glycans. Next, we focus on the impact of dietary sialic acids, a unique and essential class of monosaccharides, on the gut microbiome and human health. Sialic acids are essential to many physiological processes, play a large role in shaping both the infant and adult microbiome, and allow exploration of how minor chemical modifications in sugar structure can shape the microbiome. Although many authors have reviewed sialic acids in the past, to our knowledge a comprehensive review focusing specifically on dietary sialic acids and the gut microbiome has not been published. In the literature, “sialic acids” is often used to refer to both the group and its most common member, N-acetylneuraminic acid. In this review, we will refer to N-acetylneuraminic acid by its abbreviation Neu5Ac and reserve the term sialic acids for the group as a whole.

ialic acids are acidic 9-carbon monosaccharides, derivatives of neuraminic acid, and ubiquitous in all vertebrate glycosylation systems. Sialic acids often serve as the terminal sugars in *N*-linked and *O*-linked vertebrate glycans that decorate cell-surface proteins and lipids, and as such they are often some of the first monosaccharides encountered in cell–cell interactions.^70,71^ They play essential roles in immune system signaling, cell adhesion, membrane transport, and many other processes.^71–73^ The most abundant mammalian sialic acids are Neu5Ac and its close chemical cousin *N*-glycolylneuraminic (Neu5Gc) acid (**Figure 3A**).^71^ Humans cannot produce Neu5Gc due to loss of the CMP-*N*-acetylneuraminic acid hydroxylase (CMAH) enzyme,^83–85^ and as such Neu5Gc is perceived as a foreign antigenic sugar by the human immune system.^86,87^ Sialic acids are present in our diet in *N* – and *O*-linked glycans from animal-derived proteins, and Neu5Gc can be incorporated into human glycoconjugates following ingestion of certain animal-derived foods rich in Neu5Gc, chiefly red meat.^88,89^ Neu5Ac and Neu5Gc have drastically different effects on human health, with Neu5Ac a natural and beneficial component of human glycans and Neu5Gc an antigenic and pro-inflammatory component.^90–94^

### Sialic acid metabolism by gut bacteria

Human-associated bacteria, including gut microbiota, use sialic acids primarily as either a nutrient source or as a signaling molecule to interact with their host.^95^ For example, given the role of Neu5Ac on host cells in inhibiting autoimmune signaling through Siglec proteins,^96^ some pathogens evade the immune system by prominently displaying Neu5Ac on their cell surfaces.^97,98^ For an extensive review of sialic acids catabolism by human pathogens all over the body, we refer to ref. 87.^98^ Bacteria can synthesize sialic acids *de novo* or scavenge from the surrounding environment.^72,95^ Complete metabolism of sialic acids requires a sialidase to release the monosaccharide from the glycan, a transporter protein to transport the monosaccharide inside the cell, and a suite of intracellular enzymes to convert sialic acids into a sugar fed into different metabolic pathways (**Figure 3B**).^72^ Many common gut microbes contain genes for part of or for this entire pathway, affecting their role in the gut microbial community, and through that the community’s potential effects on human health.

The first full Neu5Ac metabolism pathway was described in *Escherichia coli* in 1999^99^ and the ability of *E. coli* to metabolize Neu5Ac has since been shown to be important for gut colonization in mice.^100^ The *Nan* gene cluster in *E. coli* encodes the sialic acids uptake transporter NanT and three catabolic enzymes (NanA lyase, NanK kinase, and NanE epimerase) that catalyze the conversion of Neu5Ac to pyruvate and *N*-acetylglucosamine-6-phosphate. This is further metabolized through the *N*-acetylglucosamine (GlcNAc) catabolic pathway (Figure 3b). Neu5Gc is transported and catabolized by *E. coli* using the same NanT transporter and NanA lyase but producing glycolate instead of pyruvate.^101^ Similar Neu5Ac catabolic gene clusters with variations in the identity of sialic acid transporter were identified in 46 out of 1,902 bacterial genomes examined in a 2009 study.^102^ However, 91% of these 46 organisms were able to colonize humans, indicating the ability to metabolize sialic acids is particularly valuable for bacteria in human-associated niches. Nine of these organisms were gut commensals (*Anaerotruncus colihominis, Dorea formicigenerans, D. longicatena, Faecalibacterium prausnitzii, Fusobacterium nucleatum, Ruminococcus gnavus, Lactobacillus sakei, L. plantarum*, and *L. salivarius*), while several others were known gut pathogens (*E. coli, Shigella* (species unspecified), *Salmonella enterica, Yersinia enterocolitica, Vibrio vulnificus*, and *V. cholerae*).^102^ A similar analysis in 2015, of 4,497 genomes in NCBI at the time, found that 5.9% of species contained genes for the full pathway of Neu5Ac metabolism; again, these organisms primarily colonize humans or animals.^98^ An alternative Neu5Ac utilization pathway was identified in the gut commensal *Bacteroides fragilis* (Figure 3b) and involves a putative sialic acid transporter from the MFS superfamily (NanT), a non-orthologous Neu5Ac lyase (NanL), and two novel catabolic enzymes, epimerase NanE3 and kinase RokA.^74^ The *nanLE2T* gene cluster from *B. fragilis* was further identified in many colonic bacteria from the *Bacteroidetes* phylum, including *B. vulgatus* and *Parabacteroides distasonis*, but not in *B. thetaiotaomicron*, which encodes a sialidase but lacks the *nanLE2T* genes to fully metabolize sialic acids.^103^ Other microbes like *Clostridioides difficile* or *E. coli* lack a sialidase but encode a complete pathway to metabolize sialic acids.^104^

**Figure 4.**
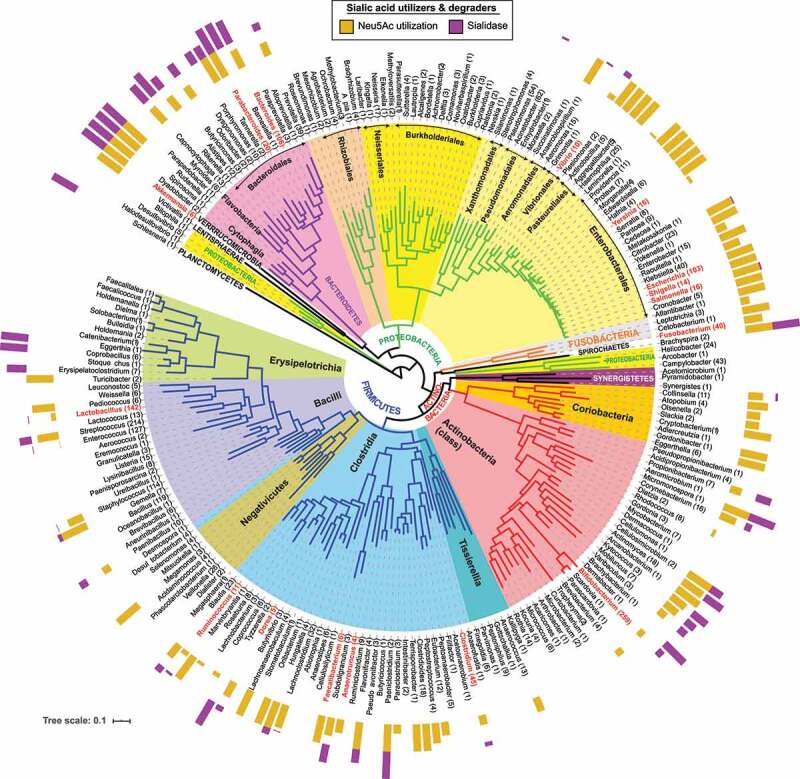
Genomic distribution of Neu5Ac utilizers and degraders in human gut microbiome strains. The phylogenetic tree was constructed using concatenated sequences of universal ribosomal proteins from approximately 2,600 human gut microbial genomes from the PATRIC genomic database^107^ by RAxML version 8,^108^ then shrunk to genus representatives and visualized via iTOL.^109^ Numbers next to genera in the outermost level of the tree indicate the number of analyzed genomes per genus. Bars adjacent to each genus indicate the proportion of genomes that contain genes for: a Neu5Ac transporter and full Neu5Ac catabolism pathway (gold bars); and one or more Neu5Ac sialidase genes (purple bars). Genera mentioned in this review are shown in red text

We analyzed the distribution of sialic acids utilization pathway and sialidase genes across a reference set of 2,662 genomes representing ~700 species and ~200 genera of bacteria from the human gut.^105^ For genomic identification of genes encoding sialidases, Neu5Ac transporters, and catabolic enzymes (Figure 3b), we used a subsystems-based approach implemented in the SEED platform.^106^ Each reference genome was assigned binary phenotypes reflecting the presence/absence of: (i) a complete Neu5Ac utilization pathway; and (ii) sialidase enzyme(s) (Figure 4). Approximately 1,040 strains were predicted as Neu5Ac-utilizing strains, representing ~80 bacterial genera. Among these, a sialidase was identified in 40% of the strains, including prominent colonic bacteria from the *Akkermansia, Bacteroides, Bifidobacterium, Clostridium, Flavonifractor, Parabacteroides*, and *Prevotella* genera. Another subgroup of strains that lack a sialidase but are capable of sialic acid utilization includes both human gut symbionts such as *Anaerococcus, Blautia, Escherichia, Eubacterium, Faecalibacterium, Fusobacterium*, and also a number opportunistic pathogens including *Clostridioides, Staphylococcus*, and *Streptococcus spp*. Finally, ~100 strains from 27 microbial genera possess a sialidase but apparently lack the sialic acid utilization capability. These include 18 *Bacteroides* strains (e.g. *B. faecis, B. intestinalis, B. thetaiotaomicron*), 6 *Porphyromonas* strains, and 6 *Coprobacillus* strains. The high prevalence of many of these strains in the gut microbiome suggests even strains that solely release Neu5Ac from underlying glycans contribute to the overall sialic acid degradation capability of gut communities.

These mixed catabolic capabilities fit with studies showing ingested complex polysaccharides can be digested and metabolized by different gut organisms, in a syntrophic or synergistic interaction network.^110^ In support of this, recent studies of *Salmonella enterica* and *C. difficile* showed that these organisms expand following antibiotic treatment through scavenging of sialic acids liberated from ingested food by other gut microbes such as *B. thetaiotaomicron*.^111^ Colonization with *B. thetaiotaomicron* lacking a sialidase inhibited *C. difficile* expansion in the mouse gut, while feeding with exogenous Neu5Ac reversed these effects.^111^ Similarly, Huang et al.^112^ showed that increased sialidase activity from *B. vulgatus* drives *E. coli* expansion in a mouse model of colitis. Hence, sialic acids released in the gut by one organism can be scavenged and metabolized by other organisms lacking a sialidase, causing effects that ripple through the metabolic network.

Although most research has been done on Neu5Ac, microbes can also act on modifications of Neu5Ac or on other sialic acids (Figure 3a). Neu5Ac modified with an *O*-acetyl group is generally resistant to release by sialidases. However, recent studies of *B. fragilis* show the *O*-acetylesterase EstA removes 9-*O*-acetyl esterifications, allowing sialidases to release these modified Neu5Ac molecules and promote *in vitro* growth of *E. coli*.^113^ Although not confirmed *in vivo* yet, this could provide another example of bacterial interactions to share metabolic capabilities. Previous studies of the commensal anaerobe *Ruminococcus gnavus* showed it cannot grow on unmodified Neu5Ac alone and instead uses an intramolecular *trans*-sialidase to release 2,7-anhydro-Neu5Ac from α2-3-linked sialic acids.^114^ 2,7-anhydro-Neu5Ac is then selectively transported across the *Ruminococcus* cell membrane and converted back to Neu5Ac for further metabolism.^115^ This strategy, which prevents other organisms from utilizing the uncommon 2,7-anhydro-Neu5Ac, seems designed to conserve resources for *R. gnavus* as opposed to the cross-talk seen in other sialic acid processing pathways. While the major part of sialidase research focuses on Neu5Ac, some recent studies have examined the activity of gut microbe sialidases on Neu5Gc. Zaramela et al.^116^ reported the discovery of Neu5Gc-preferential sialidases from the gut microbiome of the Hadza hunter-gatherer group,^4^ with four out of the five selected *Bacteroides* sialidases displaying preferential release of Neu5Gc over Neu5Ac in at least one of the tested conditions. Further exploration of metabolism of these and other sialic acid modifications will undoubtedly reveal more novel microbial strategies to harvest sialic acids.

### Dietary sialic acids, gut microbiome composition, and human health

#### Sialylated HMOs and the infant microbiome

The infant gut microbiome is thought to start developing in utero through fetal ingestion of amniotic fluid.^117^ Peri – and post-natally, the microbiota composition is heavily influenced by mode of fetal delivery (vaginal versus Cesarean section) and infant food source (breast milk versus formula).^117,118^ Human milk oligosaccharides (HMOs) represent a potent source of sialic acids (and other monosaccharides) that is unique to the infant diet (Figure 1c). HMOs are a group of over 200 oligosaccharide structures present in human breast milk, making up the third most abundant component of milk at 5–15 g/L (following lactose at 70 g/L and lipids at 40 g/L).^119^ The composition and overall amount of HMOs in breast milk varies by woman and by time since delivery.^120^ The majority of HMOs are not absorbed by the infant in the small intestine for nutrition, but instead persist into the colon where they have a significant impact on infant health (**Figure 2**).^121^ For example, HMOs have been shown to directly inhibit infant gut colonization by pathogens like enterotoxic *E. coli, V. cholerae* toxin, *Campylobacter jejuni*, rotaviruses, and noroviruses.^122–124^

HMOs in general, and sialylated HMOs (HMOs containing sialic acid) in particular, also promote the growth of particular beneficial microorganisms in the infant gut. Of the taxa studied from the infant gut microbiome, only the *Bifidobacterium* and *Bacteroides* genera have been shown to metabolize a broad range of HMOs.^125,126^ The gut microbiome of breast-fed infants is typically dominated by *Bifidobacterium*, representing up to 70% of gut microbiota in breast-fed infants compared to 31% in formula-fed infants.^127^ A study of individual gut microbes in isolation showed that the sialylated HMOs 3ʹ-sialyllactose (3’SL) and 6ʹ-sialyllactose (6’SL) specifically promoted the growth of seven *Bifidobacterium longum* strains, as well as *B. vulgatus* and *B. thetaiotaomicron*.^126^ 6’SL but not 3’SL promoted growth of *Lactobacillus delbrueckii*, although *L. rhamnosus* did not show appreciable growth on HMOs (Yu 2013).^126^ In particular, *B. longum* subsp. *infantis* is capable of fully metabolizing all HMOs studied to date and of growing on Neu5Ac alone in vitro.^128^ The *B. longum* subsp. *infantis* genome contains a 43-kb gene cluster (HMO1) with 16 glycoside hydrolases and many oligosaccharide transport proteins, as well as two sialidases, *nanH1* in the HMO1 gene cluster and *nanH2*.^118,128,129^ Intriguingly, *B. longum* subsp. *infantis* appears to transfer oligosaccharides into its cytoplasm and digests HMOs to monosaccharides within the cell;^118,130,131^ by contrast other microorganisms (e.g. *Bacteroides* and *Bifidobacterium bifidum*) are thought to break down HMOs to di-/monosaccharides extracellularly and transport these components into the cytoplasm.^118,132^

Other *B. longum* strains contain genes for specific portions of the sialic acids catabolism pathway (Table 1). *B. longum* subsp. *bifido* can release monosaccharides, including Neu5Ac, from HMOs but is unable to catabolize Neu5Ac, fucose, or N-acetylglucosamine.^129^ In contrast, *B. longum* subsp. *breve* can ferment these monosaccharides but may or may not be able to release them from HMOs, in a strain-dependent manner.^133,134^ *Bacteroides* species also have variable sialic acids metabolic capabilities (Table 1). Similar to *Bifidobacterium, B. fragilis* can cleave and fully metabolize Neu5Ac from HMOs, while *B. thetaiotaomicron* can cleave but not metabolize Neu5Ac.^103^ *Bacteroides* and most *Bifidobacterium* species metabolize HMOs through the same enzymatic pathways as host mucin glycan degradation.^103^ However, despite its facility at HMO digestion, *B. longum* subsp. *infantis* does not appear to digest host mucins.^131^ These results indicate dietary Neu5Ac in HMOs is heavily involved in shaping the infant gut microbiome by promoting colonization of *Bifidobacterium* and *Bacteroides* species, potentially laying the foundation of a life-long synergy between host and gut microbes.

**Table 1.** Summary of the ability of bacteria in the infant gut microbiome to release and metabolize the sialic acid Neu5Ac from HMOs

Genus	Species	Neu5Ac release	Neu5Ac metabolism
*Bifidobacterium*	*longum* subsp. *infantis*	**+**	**+**
	*longum* subsp. *bifido*	**+**	-
	*longum* subsp. *breve*	**-/+**	**+**
*Bacteroides*	*fragilis*	**+**	**+**
	*thetaiotaomicron*	**+**	-

As in sialic acids metabolism, studies of sialic acids and the infant microbiome focus primarily on Neu5Ac. Research on the effect of Neu5Gc on the infant microbiome is virtually nonexistent. Studies in the past have not identified Neu5Gc in human breast milk, although it is readily present in bovine milk.^127,135,136^ However, a recent study of human milk composition discovered that breast milk from all 16 mothers tested (split between women who consumed cow’s milk and dairy-free almond beverages) contained HMOs with Neu5Gc, indicating that diet-derived monosaccharides can be incorporated into breast milk HMOs.^137^ The presence of Neu5Gc in breast milk further adds another possible mechanism for the development of anti-Neu5Gc antibodies, which appears in infants within the first 6 months of life.^138^ Anti-Neu5Gc antibodies drive a process of chronic low-level inflammation called xenosialitis, which has been shown in animal models to contribute to inflammatory pathologies, such as liver cancer,^93^ atherosclerosis,^94^ and other autoimmune diseases.^96^ Other possible mechanisms for anti-Neu5Gc antibody development include the presence of Neu5Gc in commercial baby foods and exposure to Neu5Gc on the surface of bacteria like non-typeable *Haemophilus influenzae*.^90,138^

solating the impact of ingested HMOs containing Neu5Ac on the infant gut microbiome is relatively simple, arguably simpler than in adults given the stereotyped diets of infants. However, tying these changes to infant health outcomes is much more difficult. A study in 2016 provides one of the most comprehensive experimental investigations of this question. Researchers inoculated germ-free mice with a defined microbial community of 25 strains isolated from the gut microbiota of a growth-stunted Malawian infant.^139^ Mice were then fed a typical Malawian diet with or without purified sialylated bovine milk oligosaccharides. Mice receiving the oligosaccharides treatment showed significantly increased weight gain, lean mass, and long bone growth, compared to the control group (caloric intake was equivalent between the groups). These effects were not seen in germ-free mice treated with oligosaccharides, indicating the microbiome plays a critical role in the health benefits observed. Similar results were seen in gnotobiotic piglets.^139^ Intriguingly, despite the gut microbiome-dependent nature of the effects, the composition of the gut community was not significantly different between oligosaccharides and control groups after treatment. However, significant transcriptional changes were observed in *B. fragilis* and *E. coli*, including upregulation of genes in the polysaccharide utilization locus of *B. fragilis*. The researchers also noted that the two *B. longum* subsp. *infantis* strains included in the community failed to colonize in the gut community in both the treatment and control groups, although strains of *B. longum* subsp. *breve, B. bifido*, and *B. catenulatum* did colonize.^139^ This is surprising given the ubiquity of *B. longum* subsp. *infantis* in the gut microbiota of human infants and its superior abilities to digest and metabolize HMOs. However, recent research indicates the ability of bacterial strains to successfully colonize the infant gut is affected by many different factors.^140^ Follow-up studies on the mechanism of increased long bone growth with sialylated oligosaccharide treatment indicated the effect came from decreased osteoclast generation and activity, in a microbiota-dependent manner.^141^ Much work remains to be done to investigate the connections between the gut microbiome and infant health.

#### Dietary sialic acids and adult health

In contrast to studies of infants and dietary sialic acids, where studies focus on microbiome composition but often do not address direct health impacts, studies of adults and dietary sialic acids focus mainly on health impacts and rarely assess microbiome composition. The ubiquity of sialic acids in mammalian glycoconjugates gives them a role in many physiological and pathological processes, from brain development to immune regulation, infections, heart disease, and diabetes.^71^ Many of these pathological processes have been associated with hypo-sialylation, or low Neu5Ac levels, of relevant molecules. Several studies have therefore looked at the effect of exogenous Neu5Ac-feeding on disease development and progression. Neu5Ac-feeding in *apoE*^−/ –^ mice (a model of atherosclerosis through knockout of ApoE, a protein heavily involved in lipid circulation and metabolism)^142^ reduced atherosclerosis plaque area, as well as lipid liver deposition, triglyceride and cholesterol levels, and expression of inflammatory cytokines and intracellular adhesion factors in aorta endothelial cells and liver cells.^92^ In a different study, oral supplementation of the Neu5Ac precursor N-acetyl-D-mannosamine in mice on a high-fat diet (to study type II diabetes) resulted in a restoration of IgG sialylation and preserved insulin sensitivity.^143^ The mechanism of action in these studies is unknown and changes in the microbiome were not investigated in either case. However, given the established connections between the gut microbiome and atherosclerosis and diabetes^144–146^ and the impact sialic acids can have on the microbiome, an investigation of gut microbiome composition in response to Neu5Ac in these disease models would be intriguing.

The impact of dietary sialic acids on the adult gut microbiome is often difficult to tease apart, given the varied diets of adults. In 2017, researchers analyzed the gut microbiota of the Hadza people, a community living an ancestral hunter-gatherer lifestyle in Tanzania, where diet composition is determined by seasonal food availability.^4^ A longitudinal analysis revealed important modifications of the microbiome over the course of a year, following shifts between dry and wet seasons that corresponded to periods of meat – and plant-based diets, respectively. Metagenomic sequencing revealed both an increased diversity and increased number (as reads per million) of carbohydrate-active enzymes (including sialidases) in dry season samples, when the Hadza diet is dominated by meat, a food rich in sialic acids.^4^ A different study, focusing specifically on sialidases, re-analyzed the Hadza data and found specific enrichment of an organism encoding a sialidase to release Neu5Gc from glycans in the dry season samples.^116^ Since Neu5Gc is not made by humans, but is specifically enriched in red meat, this finding indicates that a Neu5Gc-metabolizing microbe becomes more abundant in the Hadza gut microbiota when levels of Neu5Gc increase in the diet.

The ability of non-human mammals to produce Neu5Gc, through the functional CMAH enzyme that humans lack, has led to a great difficulty in studying the effects of anti-Neu5Gc inflammation in animal models. However, researchers have been able to work around this through the generation of *Cmah*^−/ –^ animals that, like humans, produce only Neu5Ac.^147,148^ The presence of Neu5Gc in human glycoconjugates has been implicated in numerous disease processes, such as liver cancer and atherosclerosis.^93,94^ Neu5Gc-feeding in mouse models deficient in Neu5Gc exacerbates these diseases. Of particular interest, *Cmah*^−/ –^ knockout in a background knockout of the low-density lipoprotein receptor (*Ldlr*^−/-^) reproduces the human-specific Neu5Gc deficiency in a classic atherosclerosis model.^94^ Neu5Gc-feeding in this *Cmah*^−/ –^ *Ldlr*^−/ –^ mouse model demonstrated significantly more atherosclerosis plaque size and necrotic core volume, compared to control groups.^94^ Feeding of Neu5Gc in a *Cmah*^−/ –^ mouse model (without the *Ldlr*^−/ –^ deletion) showed distinct changes in the gut microbiome, with *Bacteroides, Barnesiella, Clostridium, Parabacteroides, Roseburia*, and *Turicibacter* significantly enriched compared to feeding with Neu5Ac.^116^ Examining the effects of Neu5Gc-feeding on the microbiome of the *Cmah*^−/ –^ *Ldlr*^−/ –^ model and potential relationships between these changes and atherosclerosis could further our current understanding of the role the gut microbiome plays in cardiovascular disease.

## Conclusion

The impact of carbohydrates on the gut microbiome is nuanced, with differences seen from alterations in large carbohydrate classes, individual monosaccharides, and even modifications of individual monosaccharides. Many studies have examined the effects of broad glycan classes, such as fiber, in animals, and humans. Many other studies have looked at the ability of bacteria common in the gut microbiome to metabolize individual monosaccharides or glycans, either in vitro or in vivo. Microbiome composition shifts rapidly and reproducibly with dietary changes, emphasizing the potential therapeutic benefits of diet modifications. However, as a genomic analysis of organisms from the Human Gut Microbiome project shows (Figure 4), we have barely scratched the surface in our studies of individual microbes that can metabolize monosaccharides like sialic acids. Studies are also lacking on the effect of dietary sialic acids on the adult gut microbiome. Given the prominent microbiome and health effects seen in infants with sialylated HMOs, we expect that dietary sialic acids could drive similarly important microbiome modifications in adults. These modifications could be a missing link to explain the changes in disease phenotypes observed with dietary sialic acids in animal models. The impact of individual dietary glycans on the gut microbiome is therefore an essential field of research as we continue to explore the relationship between the gut microbiome and human disease.
